# Prognosis of patients with whiplash-associated disorders consulting physiotherapy: development of a predictive model for recovery

**DOI:** 10.1186/1471-2474-13-264

**Published:** 2012-12-29

**Authors:** Tony Bohman, Pierre Côté, Eleanor Boyle, J David Cassidy, Linda J Carroll, Eva Skillgate

**Affiliations:** 1 Institute of Environmental Medicine, Karolinska Institutet, Box 210, Stockholm, SE-17177, Sweden; 2 University of Ontario, Institute of Technology, Faculty of Health Sciences, 2000 Simcoe Street North, Oshawa, ON, L1H 7K4, Canada; 3 Division of Epidemiology, Dalla Lana School of Public Health, University of Toronto, Health Science Building, 155 College Street, Toronto, ON, M5T 3M7, Canada; 4 Division of Health Care and Outcomes Research, Toronto Western Research Institute, University Health Network, LuCliff Place, 700 Bay Street, Suite 2201, Toronto, ON, M5G 1Z6, Canada; 5 Institute of Sports Science and Clinical Biomechanics, Faculty of Health, University of Southern Denmark, Campusvej 55, Odense M, 5230, Denmark; 6 School of Public Health, University of Alberta, 4075 RTF 8308-114 Street, Edmonton, AB, T6G 2E1, Canada; 7 Skandinaviska Naprapathögskolan (Scandinavian College of Naprapathic Manual Medicine), Kräftriket 23A, Stockholm, SE-11419, Sweden

**Keywords:** Prediction, Prognosis, Whiplash-associated disorders, Neck pain, Physical therapy, Cohort, Recovery, Regression, Discrimination

## Abstract

**Background:**

Patients with whiplash-associated disorders (WAD) have a generally favourable prognosis, yet some develop longstanding pain and disability. Predicting who will recover from WAD shortly after a traffic collision is very challenging for health care providers such as physical therapists. Therefore, we aimed to develop a prediction model for the recovery of WAD in a cohort of patients who consulted physical therapists within six weeks after the injury.

**Methods:**

Our cohort included 680 adult patients with WAD who were injured in Saskatchewan, Canada, between 1997 and 1999. All patients had consulted a physical therapist as a result of the injury. Baseline prognostic factors were collected from an injury questionnaire administered by Saskatchewan Government Insurance. The outcome, global self-perceived recovery, was assessed by telephone interviews six weeks, three and six months later. Twenty-five possible baseline prognostic factors were considered in the analyses. A prediction model was built using Cox regression. The predictive ability of the model was estimated with concordance statistics (c-index). Internal validity was checked using bootstrapping.

**Results:**

Our final prediction model included: age, number of days to reporting the collision, neck pain intensity, low back pain intensity, pain other than neck and back pain, headache before collision and recovery expectations. The model had an acceptable level of predictive ability with a c-index of 0.68 (95% CI: 0.65, 0.71). Internal validation showed that our model was robust and had a good fit.

**Conclusions:**

We developed a model predicting recovery from WAD, in a cohort of patients who consulted physical therapists. Our model has adequate predictive ability. However, to be fully incorporated in clinical practice the model needs to be validated in other populations and tested in clinical settings.

## Background

Whiplash injuries result from an acceleration-deceleration mechanism to the neck, usually following a traffic collision and whiplash-associated disorders (WAD) describes the symptomatology related to these injuries. WAD includes neck pain, headache, arm pain and other physical complaints [1]. The pathophysiology of WAD is not well understood, but its aetiology likely combines physical and psychological causes [2].

The annual incidence of WAD in Western countries is estimated to be at least 300 per 100,000 inhabitants [3]. WAD is associated with a significant financial burden to society. In the United States and Europe the annual cost of WAD was estimated to be $3.9 billion and $13.4 billion respectively [4,5]. Physical therapy is a common health care option for persons with neck pain, including WAD, and a large proportion of patients consulting physical therapists have neck pain [6-8].

Although the prognosis of WAD is generally favourable, previous studies have found as much as 50% of the affected individuals to be symptomatic one year after the injury [5,9]. To date, predicting the outcome of WAD remains challenging and few clinical prediction tools exists to assist health care providers in establishing the prognosis for patients. Searching the literature, we found only one prediction model developed for patients with WAD [10]. The authors found increasing age, number of initial physical symptoms, initial upper back pain, upper extremity numbness and vision disturbances to predict persistent symptoms six months post-injury in patients who presented to emergency departments [10].

Post-injury symptoms such as pain and disability are well-established prognostic factors for the recovery from WAD [9,11,12]. In addition there is evidence that psychological factors such as recovery expectations, pain catastrophizing and depression predict poor recovery [9,11,13]. However, there is conflicting evidence for the prognostic value of prior pain, prior health and comorbidities as well as for socio-demographic variables [9,11,12].

It is important for health care providers such as physical therapists to predict which patients with WAD are more likely to make a successful recovery. This knowledge can improve the care of patients with WAD and help manage their expectations. It is recommended that prediction tools be developed in well-defined patient population so that their application is generalizable to similar populations. Therefore, different prediction tools need to be developed for patient groups that consult different health care providers [14,15]. The purpose of our study was to develop a predictive model for the recovery of WAD in a sample of patients who consulted physical therapists within six weeks of their injury.

## Methods

### Design and study population

**Figure 1:**
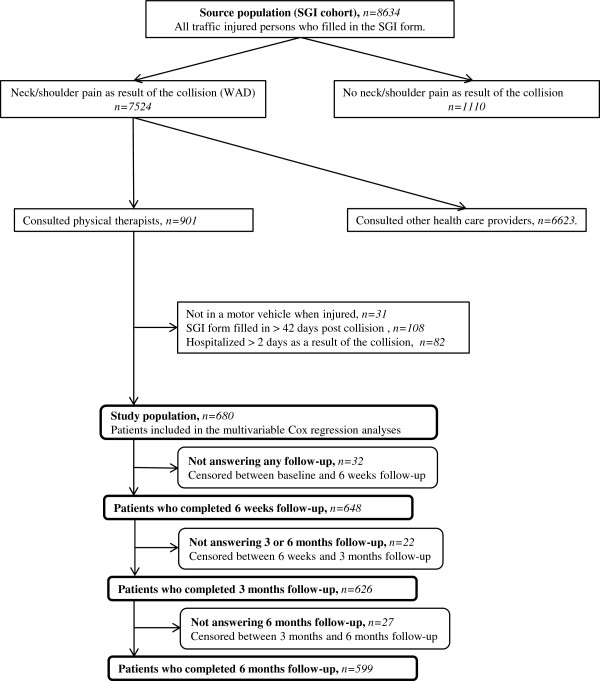
**Inclusion process and progress of patients in the study population.** WAD: Whiplash-associated disorders. SGI: The Saskatchewan Government Insurance.

In this study we used data from the Saskatchewan Government Insurance (SGI) study, a population-based inception cohort study of 8634 individuals injured in a traffic collision in the province of Saskatchewan, Canada, between December 1, 1997 and November 30, 1999 [16-19]. Eligible participants for the SGI study were residents of Saskatchewan, 18 years of age and older who reported their collision to the SGI. This included all individuals who consulted with a health care provider for their injury, because providers were mandated to inform SGI of all traffic injuries. Excluded from the SGI cohort were Workers Compensation claims since those are covered under a different system, individuals unable to participate due to lack of English language and individuals with serious unassociated illness. The sample used in this analysis is a sub-cohort of the SGI study. The sample includes patients with WAD who consulted with a physical therapist (PT) between the date for the collision and the date for reporting to SGI. Patients with WAD were defined by answering “Yes” to the question “Did the accident cause neck or shoulder pain?” Excluded were patients who were not in a motor vehicle when injured and patients reporting their injury to SGI more than 42 days after the collision. We also excluded patients hospitalized for more than 2 days after the injury since this indicates a more severe trauma (Figure 1).

### Data collection

#### Prognostic factors for the predictive model

Potential prognostic factors were collected from the baseline study questionnaire included in the SGI form filled in by the patient. The study questionnaire included items on socio-demographics, injury-related pain intensity and location, activity limitations, comorbid health conditions, pre- and post-injury general health, health care provision, depressive symptomatology and work status. We selected potential prognostic factors based on the literature and clinical experience of the authors (Table 1) [9,11,12,20]. The selected factors were grouped into three domains that represented the sequential approach of the medical history obtained by a physical therapist during an initial consultation. The rationale for using this approach was to determine if expanding the breadth of information collected during the medical history improve the ability of physical therapists to predict recovery. The three domains were:

1) *Socio-demographics:*

Age, sex, marital status (single, married, widowed, separated/divorced), education (< grade 8, grade 8, high school, post-secondary school, technical school, university) and work status (not working; unemployed, disability leave, maternity leave, retired, homemaker/working; fulltime, part time, student).

2) *Collision, symptoms, comorbidity and health care:*

Number of days to reporting the collision (0-42 days), average pain intensity, reported at baseline, in neck/shoulder, low back and of headache. Pain intensity was measured on an 11-point Numeric rating scale (NRS: 0-10) where “0” means “no” pain and “10” means “pain as bad as it could be”. The NRS is a reliable and valid method for assessing pain in various patient categories [21,22]. According to the optimal cut-points for classifying neck pain intensity of 4/7, suggested by Fejer and colleagues, neck/shoulder pain intensity (from now denoted as neck pain intensity) was categorized into no (0), mild (1-4), moderate (5-7) and severe (8-10) pain [23]. The same categories were used for headache and back pain intensity as we only found varying cut-points for these measures in literature [24,25]. Other factors in this domain were; pain other than neck and back pain, feeling of numbness, tingling or pain in arms or hands, pain when moving the neck, reduced neck movement and sleeping problems. All assessed by the answer “Yes” or “No”. Musculoskeletal problems and headache affecting health within 6 months before the collision were categorized as absent, no/mild effect or severe effect on health. Activity restrictions were measured by answering “Yes” to the question: “Have the injuries prevented you from carrying out any of the following activities?”; daily home activities, leisure activities (activities not related to home, work or education). Finally the number of self-reported visits to PT and medical doctors (MD) since collision was considered.

3) *General health and psychology:*

Baseline expectations of recovery was ascertained by asking; “Do you think your injury will…”, with the response categories of; “Get better soon”, “Get better slowly”, “Never get better” and “Don^′^t know”. Current and pre-collision general health was assessed by using an item from the Short Form 36; “In general, how would you say your health is now?” and “How was your health the month before the collision?” (Excellent, Very good, Good, Fair and Poor) [26]. We determined if the collision caused anxiety or worry by the answer “Yes” or “No”. Finally, information about depressive mood in the past week was assessed with the 20-item Centre for Epidemiologic Studies Depression Scale (CES-D). The scale is a reliable and valid instrument for measuring depressive symptomatology in both healthy and ill populations. We used a cut-point of 16, recommended for population-based studies, with scores of 16 or above indicating depressed mood [27-29].

**Table 1:** Baseline characteristics of the study population and the “complete study sample”

Characteristics (potential prognostic factors)^*^	**Category**	**Study population (n=680)**			Complete study sample^**^(n=569)	
		**Freq.**^**a**^**(n)**	**%**^**b**^	**Miss.**^**c**^**(n)**	**Freq.**^**a**^**(n)**	**%**^**b**^
***Socio-demographics***						
Age, mean (SD)		39	(15)		39	(15)
Sex	Females	471	69.3		403	70.8
Marital status	Single	215	31.6		188	33.0
	Married	381	56.0		315	55.4
	Widowed	21	3.1		18	3.2
	Separated/Divorced	63	9.3		48	8.4
Education	< Grade 8	33	4.9	1	28	4.9
	Grade 8	97	14.3		76	13.4
	High school	173	25.4		148	26.0
	Post-secondary school	189	27.8		165	29.0
	Technical school	102	15.0		83	14.6
	University	85	12.5		69	12.1
Work status^d^	Working	537	79.0		452	79.4
***Collision, symptoms, comorbidity and health care***						
No. of days to reporting the collision, mean (SD)		16	(10)		16	(9)
Neck pain intensity^e^	No pain	0	0		0	0
	Mild	91	13.4	12	85	14.9
	Moderate	300	44.1		260	45.7
	Severe	277	40.7		224	39.4
Low back pain intensity^e^	No pain	295	43.4	12	259	45.5
	Mild	79	11.6		66	11.6
	Moderate	171	25.2		143	25.1
	Severe	123	18.1		101	17.8
Headache intensity^e^	No pain	116	17.1	3	105	18.4
	Mild	101	14.9		82	14.4
	Moderate	233	34.3		201	35.3
	Severe	227	33.4		181	31.8
Pain other than neck and back pain	Yes	529	77.8		432	75.9
Symptoms in arms or hands^f^	Yes	293	43.1		235	41.3
Pain when moving neck	Yes	593	87.2		494	86.8
Reduced neck movement	Yes	525	77.2		437	76.8
Sleeping problems	Yes	470	69.1		396	69.6
Musculoskeletal problem before collision^g^	Absent	459	67.5	3	384	67.5
	No/Mild	140	20.6		125	22.0
	Severe	78	11.5		60	10.5
Headache before collision^h^	Absent	412	60.6	1	345	60.6
	No/Mild	180	26.5		154	27.0
	Severe	87	12.8		70	12.4
Restrictions in daily home activity	Yes	411	60.4		342	60.1
Restrictions in leisure activity^i^	Yes	114	16.8		99	17.4
MD visits since collision^j^	1	285	41.9	5	251	44.1
	2	238	35.0		192	33.7
	≥ 3	152	22.4		126	22.1
PT visits since collision^k^	1	246	36.2	10	198	34.8
	2	189	27.8		164	28.8
	≥ 3	235	34.6		207	36.4
***General health and psychology***						
Recovery expectations^l^	Better soon	142	20.9	1	130	22.9
	Better slowly/Never better	320	47.1		267	46.9
	Don′t know	217	31.9		172	30.2
Current general health^m^	Excellent	17	2.5		15	2.6
	Very good	54	7.9		49	8.6
	Good	190	27.9		164	28.8
	Fair	288	42.4		241	42.4
	Poor	131	19.3		100	17.6
General health the month before collision^n^	Excellent	255	37.5		214	37.6
	Very good	250	36.8		218	38.3
	Good	130	19.1		100	17.6
	Fair/Poor	45	6.6		37	6.5
Anxiety or worry	Yes	285	41.9		238	41.8
Depressed mood^o^	Yes	300	44.7	18	255	44.8

^*^Note: *All characteristics (potential prognostic factors) concerned problems that participants experienced as a result of the injury unless otherwise stated.*

^**^ Sample without patients who was lost to follow-up (n=81) and/or had missing data on potential prognostic factors (n=47).

^a^ No. of subjects in the category unless otherwise stated.

^b^ Percent unless otherwise stated.

^c^ No. of missing answer for the potential prognostic factor.

^d^ Work status: Not working; unemployed, disability leave, maternity leave, retired, homemaker, Working; fulltime, part time and student.

^e^ 11 point numeric rating scale (NRS) were 0 = no pain at all and 10 = pain as bad as it could be. Mild: 1-4, Moderate: 5-7, Severe: 8-10.

^f^ Feeling of numbness, tingling or pain in arms or hands.

^g^ The effect on health from muscle, bone or joint problems within 6 months before the collision.

^h^ The effect on health from headache within 6 months before the collision.

^i^ Activities not related to home, work or education.

^j^ MD: Medical doctor.

^k^ PT: Physical therapist.

^l^ The answer to the “question”: “Do you think that your injury will…”.

^m^ The answer to the question: “In general, how would you say your health is now?”.

^n^ The answer to the question: “How was your health the month before the collision?”.

^o^ Based on the Center for Epidemiologic Studies Depressive scale (CED-S) indicating depressed mood the past week: Yes = depressed mood (CED-S ≥16).

#### Outcome

All patients were followed by telephone interviews six weeks and three, six, nine and 12 months after their collision. In the current study, we restricted our analyses to data collected up to the six-month follow-up because it corresponds to the period where maximal clinical improvement is expected [5]. The follow-up interviews provided information on self-rated recovery, pain location, pain intensity, disability, health-related quality of life, exercise, activity limitation, health care provision, depressive symptoms and work status. We used self-reported recovery from WAD as our outcome, measured with the global recovery question: “How well do you feel that you are recovering from your injuries”? Patients answering “All better (cured)” or “There has been quite a bit of improvement” were defined as recovered. Not recovered was equal to answer; “There has been some improvement”, “There has been no improvement”, “I am getting a little worse” or “I am getting much worse”. This question has been shown to have adequate reliability and validity for use in epidemiological studies of WAD [18,19,30]. Time to recovery was defined at the first follow-up where the patients were defined as recovered.

### Statistical analysis

We used the Kaplan-Meier method to describe the median time to recovery of our sample. Patients who were lost to follow-up were censored at the mid-point between the last completed follow-up and the next follow-up time.

Further analysis included three phases;

#### Univariate phase

Step 1

We performed a univariate Log-rank test (LRT) for equality of survivor functions on all potential prognostic factors. Factors with p-value ≤ 0.2 were considered for the following multivariable analyses [31,32].

Step 2

Correlation between all potential prognostic factors were tested to assess collinearity. Collinearity was deemed to be present if the Spearman pairwise correlation was greater than 0.5 [33]. The presence of collinearity was managed by eliminating from the analyses the factor that was judged to be the least important from a clinical perspective.

Step 3

The proportionality assumption for each prognostic factor was verified using Schoenfeld residuals against time [31,34].

Step 4

The presence of clinically relevant statistical interactions between neck pain intensity and recovery expectations or depression were tested [9,17]. An interaction significant at p ≤ 0.05 was included in further analyses [31].

#### Multivariable phase - developing the predictive models

We used a manual backward selection procedure based on the Cox′s proportional hazard regression to build the models (described below) [31]. The strategy included the development of three models with possible prognostic factors from the domains representing the sequential gathering of medical history done by a physical therapist. The associations between a prognostic factor and recovery was reported as beta coefficients (β) with standard error (SE) and hazard rate ratios (HRR) with 95% confidence interval (95% CI).

Model 1

Factors from the domain *“socio-demographics”* were considered. In the selection procedure the factor with the highest p-value was excluded one by one until all prognostic factor themselves (or at least one category) had a p-value of < 0.1 [32,35]. The likelihood ratio test statistics was used to compare the model before and after exclusion of a factor [34,36]. A p-value of more than 0.05 indicated that excluding the variable did not significantly change the fit of the model.

Model 2

Significant factors from model 1 and factors related to *“collision, symptoms, comorbidity and health care”* were combined using the same method as for building model 1.

Final prediction model 3

Included the remaining factors from model 2 and the *“general health and psychology”* variables and were constructed using the methodology described above. Signs of collinearity were assessed using the variance inflation factor of more than 10 as the criteria for collinearity. Presence of collinearity was handled as in the univariate phase.

#### Evaluation phase

The predictive ability of the models was measured with the Harrell′s concordant statistics (c-index) with 95% CI. A model with a c-index of 0.5 has no predictive ability while a c-index of 1.0 indicates perfect predictive ability [34]. The internal validity of the final model was checked by cross validating 500 bootstrap replicate to get bias corrected c-index with 95% CI [34,37]. This c-index indicates the predictive ability of the model in similar WAD populations as in this study. We assessed overfitting by computing the shrinkage factor [34,37]. A shrinkage factor of 1.0 indicates perfect fit of the model while a factor of for example 0.8 indicates that 20% of the inference is due to overfitting. The overall goodness of fit of the final model was assessed by plotting Cox-Snell residuals and by computing a score test based on the Martingale residuals [31,34].

#### Sensitivity analyses

To evaluate the impact of missing data we repeated the univariate and multivariable analyses with a “complete study sample”. In this sample we excluded patients who were lost to follow-up and/or with missing data on potential prognostic factors.

All p-values were two-sided and analyses were completed using SAS version 9.3 TS level 1MO (Cary, NC: SAS Institute) and Stata/IC version 12.1 (Stata Corp LP, USA). This study was approved by the University Health Network Research Ethics Board (REB 10-0216-AE), Toronto, Ontario, Canada. The original inception cohort study was approved by the Research ethics Boards of University of Saskatchewan and the University of Alberta [19].

## Results

### Study population

**Figure 2:**
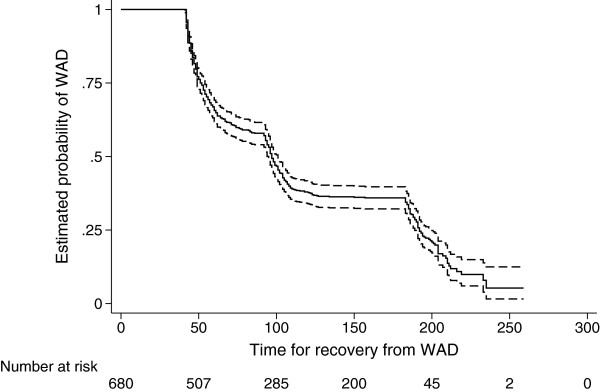
**Kaplan-Meier survival curve for recovery from whiplash-associated disorders (WAD) (n=680).** Solid line: Estimated probability of WAD. Dashed lines: 95% Confidence Interval. Risk table showing number of patients at “risk” for recovery from WAD during the follow-up period.

Our study sample included 680 patients with WAD consulting a PT (Figure 1). All patients consulting a PT had also visited a medical doctor (MD). Eighty-eight percent of the patients (n=599) completed the follow-up at 6 months (Figure 1). Table 1 lists the baseline characteristics for the study population and the sample of patients with complete data. There were no major differences in characteristics between these two samples. The mean age of our population was 39 years (Standard Deviation: 15) and 69.3% were females. Almost half of the patients (44.6%) had graduated from high school and the majority (79.0%) were working. All patients reported neck/shoulder pain as a result of the collision (according to our inclusion criteria for WAD) and had a mean baseline neck pain intensity of 6.8/10 (SD: 2.0). Low back pain and headache as a result of the collision were reported by 54.9% and 82.6% of the study population respectively. Most patients (66.7%) believed that they should get better, either soon or slowly, while 1.2% felt they would never get better and the rest did not know. Fair or poor general health the months before the collision were reported by 6.6% of the patients while 61.7% reported fair or poor general health at baseline. Median time between collision and baseline was 14 days and the mean number of MD and PT visits during that time period was 1.9 (SD 1.1) and 2.6 (SD 2.1) respectively. At the six months interview, 484 subjects (71.2%) had recovered with a median time to recovery of 97 days (Figure 2).

### Predictive model

#### Univariate phase

All potential prognostic factors except marital status, work status and pain when moving the neck met the LRT criteria to be included in the multivariable phase. We found no statistical significant interactions, bivariate correlations or violations of the proportionality assumption.

#### Multivariable phase

The results of the sequential backward selection process for the three models are presented in Table 2. The final prediction model 3 included; age, number of days to reporting the collision, neck pain intensity, low back pain intensity, pain other than neck and back pain, headache before collision and recovery expectations. Neck pain intensity and current general health showed signs of collinearity. Therefore, we excluded current general health from the multivariable analyses as we considered it to be less clinically relevant than neck pain intensity.

**Table 2:** **Results from the multivariable analyses of recovery from WAD**^**a**^**, c-index and internally validated c-index (n=680)**

**Prognostic factors**	**Model 1**^**a**^**(n=679)**^**b**^	**Model 2**^**a**^**(n=648)**^**b**^	**Final model 3**^**a**^**(n=633)**^**b**^		
	**β** (SE)	**β** (SE)	**β** (SE)	**HRR** (95% CI)	**p-value**
**Age**	**-0.01** (0.00)	-**0.01** (0.00)	**-0.01** (0.00)	**0.99** (0.98, 1.00)*	0.002
**Education**					
< Grade 8^**^	**0.0**	**0.0**	**-**	-	-
Grade 8	**0.21** (0.28)	**0.23** (0.28)	**-**	-	-
High school	**0.51** (0.27)	**0.48** (0.27)	**-**	-	-
Post-secondary school	**0.28** (0.27)	**0.29** (0.27)	**-**	-	-
Technical school	**0**.**41** (0.28)	**0.31** (0.28)	**-**	-	-
University	**0.69** (0.28)	**0.59** (0.28)	**-**	-	-
**No. of days to reporting the collision**		**-0.02** (0.01)	**-0.02** (0.01)	**0.98** (0.97, 0.99)	≤0.001
**Neck pain intensity**^**c**^					
Mild^**^		**0.0**	**0.0**	**1.0**	
Moderate		**-0.48** (0.14)	**-0.43** (0.14)	**0.65** (0.50, 0.85)	0.002
Severe		**-0.70** (0.15)	**-0.50** (0.15)	**0.61** (0.45, 0.82)	0.001
**Low back pain intensity**^**c**^					
No pain^**^		**0.0**	**0.0**	**1.0**	
Mild		**-0.01** (0.16)	**0.17** (0.15)	**1.19** (0.88, 1.61)	0.26
Moderate		**-0.29** (0.12)	**-0.16** (0.12)	**0.85** (0.68, 1.07)	0.17
Severe		**-0.46** (0.15)	**-0.41** (0.15)	**0.66** (0.49, 0.89)	0.01
**Pain other than neck and back pain**					
No^**^		**0.0**	**0.0**	**1.0**	
Yes		**-0.38** (0.11)	**-0.35** (0.11)	**0.71** (0.57, 0.88)	0.002
**Musculoskeletal problem before collision**^**d**^					
Absent^**^		**0.0**	**-**	-	-
No/Mild		**-0.03** (0.12)	**-**	-	-
Severe		**-0.33** (0.18)	**-**	-	-
**Headache before collision**					
Absent^**^		**0.0**	**0.0**	**1.0**	
No/Mild		**0.32** (0.11)	**0.28** (0.11)	**1.32** (1.07, 1.63)	0.01
Severe		**-0.01** (0.15)	**-0.03** (0.15)	**0.97** (0.72, 1.31)	0.87
**MD visits since collision**^**e**^					
1^**^		**0.0**	**-**	**-**	-
2		**-0.05** (0.11)	**-**	-	-
≥ 3		**-0.26** (0.13)	**-**	-	-
**Recovery expectations**^**f**^					
Better soon^**^			**0.0**	**1.0**	
Better slowly/Never better			**-0.66** (0.12)	**0.51** (0.41, 0.65)	≤0.001
Don′t know			**-1.09** (0.14)	**0.34** (0.26, 0.44)	≤0.001
**C-index (95% CI)**	**0.56** (0.53, 0.59)	**0**.**65** (0.63, 0.68)	**0.68** (0.65, 0.71)		
**Internal validated c-index (95% CI)**	**0.55** (0.51, 0.58)	**0**.**63** (0.59, 0.66)	**0.67** (0.63, 0.70)		

Note: Overall Goodness of fit for the final model was adequate according to the Cox-Snell residual plot and the score test (p= 0.66).

^*^ 95% CI: 0.984, 0.996.

^**^ Reference category.

^a^ Cox proportional hazard regression: Model 1: Result from including socio-demographics related factors in the backward selection process. Model 2: Result using prognostic factors remaining from model 1 plus factors related to collision, symptoms, comorbidity and health care. Final model 3: Result using prognostic factors remaining from model 2 plus factors related to general health and psychology.

^b^ Numbers of subjects are less than the study population (n=680) due to missing answer for prognostic factors in the backward selection procedures.

^c^ 11 point numeric rating scale (NRS) were 0 = no pain at all and 10 = pain as bad as it could be. Mild: 1-4, Moderate: 5-7, Severe: 8-10.

^d^ The effect on health from muscle, bone or joint problems within 6 months before the collision.

^e^ MD: Medical doctor.

^f^ The answer to the “question”: “Do you think that your injury will…”.

#### Evaluation phase

The predictive ability (c-index) increased for each model and reached an acceptable level of 0.68 (95% CI: 0.65, 0.71) for the final model [38]. Internal validation (c-index: 0.67 (95% CI: 0.63, 0.70)) showed a robust final model with acceptable ability to predict self-reported recovery from WAD in similar populations of WAD patients. The Cox-Snell residual plots, the score test (p=0.66) and a shrinkage factor of 0.93 all indicated that the final model 3 was robust and had a good fit.

#### Sensitivity analyses

We rebuilt our three models using the sample of patients with complete data (n=569) resulting in a final model 3 with the same prognostic factors as in the main analyses. The c-index for the final model 3 in the sensitivity analyses was 0.67 (95% CI: 0.64, 0.70).

## Discussion

We developed and internally validated a predictive model for recovery among patients with WAD consulting physical therapists within six weeks after the injury. The model has acceptable predictive ability.

Information about prognostic factors incorporated in the model is easily gathered in the medical history taken by a physical therapist. Furthermore, our results indicate that expanding the breadth of information from the medical history improves the ability to predict recovery from WAD. Patients with WAD frequently seek physical therapists and this model may be an important tool to help physical therapists in their management of these patients.

### Comparison with previous literature

To the best of our knowledge, our study is the first to develop a predictive model of recovery from WAD in patients who consulted a physical therapist. Therefore we cannot compare the developed predictive model with previous prediction studies.

Our final predictive model includes seven prognostic factors. Four of these factors (neck pain intensity, low back pain intensity, pain other than neck and back pain and recovery expectations) have previously been reported to be independent prognostic factors of recovery from WAD [9,11,12]. According to the “2000-2010 Bone and Joint Decade Task Force on Neck Pain and Its Associated Disorders”, the available evidence varies about the prognostic role of age and prior headaches [9]. Our results agree with those of Kamper and colleagues who reported that the rate of recovery from WAD is faster within the first few weeks after the collision and then slows down (Figure 2) [5]. Considering this, it seems reasonable that the factor “number of days to reporting the collision”, reflecting duration of neck pain, is one of the predictors in our final model. Although there is evidence from other studies that depressed mood predicts poor recovery in WAD, our final model did not include this factor [9,11,16]. It is possible that the physical therapists’ active clinical management of these patients attenuates the effect of depressed mood on recovery.

### Strengths and limitations

Our study meets the criteria for an optimal design of prediction studies of WAD and physical therapy [39,40]. First, we used a sample recruited shortly after the injury. Second, we clearly described our study population and prognostic factors. Our sample was population-based. All Saskatchewan health care providers were mandated to report whiplash injuries to SGI. Third, we used a long enough follow-up period for the outcome to develop. Fourth, we selected potential prognostic factors based on published evidence and clinical relevance with acceptable psychometric properties of the measurements used. Fifth, the patients were blinded to the study objective. Sixth, we used bootstrap to cross validate our model. Finally, our sample size was large enough for the numbers of associations tested in the multivariate analyses (≥ 10 events/association) [34,40]. Our follow-up rate was 88% and there were only minor differences between the study population and “the complete sample”. Furthermore, the sensitivity analyses did not differ to the main analyses. This provides confidence that attrition was not associated with our outcome of interest.

Because all patients in our sample consulted a physical therapist and a medical doctor, our prediction model should be restricted to this population. We do not know if the model is valid for patients who consulted other health care providers such as chiropractors and massage therapists [14,15].

Our study also has limitations. We did not have information about the specific treatment received by the patients prior to baseline. Treatment may have the potential to change recovery rate and if it is an important prognostic factor, this information could have improved the predictive ability of the model. Information on lifestyle factors such as e.g. physical activity, smoking and alcohol consumption were not collected with the baseline questionnaire. Moreover, self-reported measures of pain and other comorbidities prior to collision tend to be under reported by patients with post-collision neck pain, something that maybe affected our predictive model [41]. We used the data collected in the baseline questionnaire as a proxy for medical history collected by a physical therapist. It is possible that patients would answer differently when consulting a physical therapist. Therefore the predictive ability of the predictors considered in our study may have been influenced by the methods used to collect the data.

Despite these limitations, we believe the findings of this study are sound and can be reproduced.

## Conclusions

This predictive model for recovery from WAD among patients consulting physical therapy has an acceptable predictive ability, is robust and has a good fit. Our model can guide physical therapists to assess medical history information that are important for predicting recovery from WAD. Furthermore, our result can give researchers some useful information for future studies on the prognosis of WAD. Our study is the first step (derivation) in the development of a prediction rule. We recommend that our model be tested in different WAD populations (external validation). Similarly, the model needs to be tested in clinical settings to determine its impact on practice pattern, outcome and costs of care for patients with WAD (impact analysis) [42].

## Abbreviations

WAD: Whiplash-associated disorders; SGI: The Saskatchewan Government Insurance; PT: Physical therapist; MD: Medical doctor; NRS: Numeric rating scale; CES-D: Centre for Epidemiologic Studies Depression Scale; SE: Standard error; SD: Standard deviation; HRR: Hazard rate ratio; CI: Confidence Interval; LRT: Log-rank test.

## Competing interests

The authors declare that they have no competing interest.

## Authors’ contributions

The authors TB, PC, EB and ES have made substantial contributions to the design of the study, analyses and interpretation of the data and drafting of the manuscript. TB made the majority of analyses and wrote the first manuscript version. JDC was the principle investigator of the original study, and he and LJC oversaw the data collection and the formation of the databases used in our analyses [19]. All authors have critically revised and approved the final manuscript.

## Pre-publication history

The pre-publication history for this paper can be accessed here:

http://www.biomedcentral.com/1471-2474/13/264/prepub

## References

[B1] Spitzer WO Skovron ML Salmi LR Cassidy JD Duranceau J Suissa S Zeiss E Scientific monograph of the Quebec task force on whiplash-associated disorders: redefining "whiplash" and its management Spine 1995 20 1S 73S 10.1097/00007632-199501000-00001 7604354

[B2] Curatolo M Bogduk N Ivancic PC McLean SA Siegmund GP Winkelstein BA The role of tissue damage in whiplash-associated disorders: discussion paper 1 Spine 2011 36 S309 S315 22020601 10.1097/BRS.0b013e318238842a PMC3248632

[B3] Holm LW Carroll LJ Cassidy JD Hogg-Johnson S Cote P Guzman J Peloso P Nordin M Hurwitz E van der Velde G Carragee E Haldeman S The burden and determinants of neck pain in whiplash-associated disorders after traffic collisions: results of the bone and joint decade 2000-2010 task force on neck pain and its associated disorders J Manipulative Physiol Ther 2009 32 S61 S69 10.1016/j.jmpt.2008.11.011 19251076

[B4] Conlin A Bhogal S Sequeira K Teasell R Treatment of whiplash-associated disorders–part I: Non-invasive interventions Pain Res Manag 2005 10 21 32 15782244 10.1155/2005/503704

[B5] Kamper SJ Rebbeck TJ Maher CG McAuley JH Sterling M Course and prognostic factors of whiplash: a systematic review and meta-analysis Pain 2008 138 617 629 10.1016/j.pain.2008.02.019 18407412

[B6] Di Fabio RP Boissonnault W Physical therapy and health-related outcomes for patients with common orthopaedic diagnoses J Orthop Sports Phys Ther 1998 27 219 230 9513868 10.2519/jospt.1998.27.3.219

[B7] Jette DU Jette AM Physical therapy and health outcomes in patients with spinal impairments Phys Ther 1996 76 930 941 discussion 942-935 8790272 10.1093/ptj/76.9.930

[B8] Bassols A Bosch F Banos JE How does the general population treat their pain? A survey in Catalonia, Spain J Pain Symptom Manage 2002 23 318 328 10.1016/S0885-3924(01)00415-8 11997201

[B9] Carroll LJ Holm LW Hogg-Johnson S Cote P Cassidy JD Haldeman S Nordin M Hurwitz EL Carragee EJ van der Velde G Peloso P Guzman J Course and prognostic factors for neck pain in whiplash-associated disorders (WAD): results of the bone and joint decade 2000-2010 task force on neck pain and its associated disorders J Manipulative Physiol Ther 2009 32 S97 S107 10.1016/j.jmpt.2008.11.014 19251080

[B10] Hartling L Pickett W Brison RJ Derivation of a clinical decision rule for whiplash associated disorders among individuals involved in rear-end collisions Accid Anal Prev 2002 34 531 539 10.1016/S0001-4575(01)00051-3 12067116

[B11] Sterling M Does knowledge of predictors of recovery and nonrecovery assist outcomes after whiplash injury? Spine 2011 36 S257 S262 22020621 10.1097/BRS.0b013e31823881bc

[B12] Walton DM Pretty J Macdermid JC Teasell RW Risk factors for persistent problems following whiplash injury: results of a systematic review and meta-analysis J Orthop Sports Phys Ther 2009 39 334 350 19411766 10.2519/jospt.2009.2765

[B13] Carroll LJ Beliefs and expectations for recovery, coping, and depression in whiplash-associated disorders: lessening the transition to chronicity Spine 2011 36 S250 S256 22020620 10.1097/BRS.0b013e31823881a4

[B14] Beattie P Nelson R Clinical prediction rules: what are they and what do they tell us? Aust J Physiother 2006 52 157 163 10.1016/S0004-9514(06)70024-1 16942450

[B15] Cote P Cassidy JD Carroll LJ The treatment of neck and low back pain: who seeks care? who goes where? Med Care 2001 39 956 967 10.1097/00005650-200109000-00006 11502953

[B16] Carroll LJ Cassidy JD Cote P The role of pain coping strategies in prognosis after whiplash injury: passive coping predicts slowed recovery Pain 2006 124 18 26 10.1016/j.pain.2006.03.012 16644133

[B17] Carroll LJ Holm LW Ferrari R Ozegovic D Cassidy JD Recovery in whiplash-associated disorders: Do You Get what You expect? J Rheumatol 2009 36 1063 1070 10.3899/jrheum.080680 19228657

[B18] Carroll LJ Jones DC Ozegovic D Cassidy JD How well are you recovering? The association between a simple question about recovery and patient reports of pain intensity and pain disability in whiplash-associated disorders Disabil Rehabil 2012 34 45 52 10.3109/09638288.2011.587085 21936737

[B19] Cassidy JD Carroll LJ Cote P Frank J Does multidisciplinary rehabilitation benefit whiplash recovery?: results of a population-based incidence cohort study Spine 2007 32 126 131 10.1097/01.brs.0000249526.76788.e8 17202903

[B20] Kasch H Qerama E Kongsted A Bendix T Jensen TS Bach FW Clinical assessment of prognostic factors for long-term pain and handicap after whiplash injury: a 1-year prospective study Eur J Neurol 2008 15 1222 1230 10.1111/j.1468-1331.2008.02301.x 18803651

[B21] Von Korff M Jensen MP Karoly P Assessing global pain severity by self-report in clinical and health services research Spine 2000 25 3140 3151 10.1097/00007632-200012150-00009 11124730

[B22] Jensen MP Karoly P Turk DC, Melzack R Self-reported scales and procedures for assessing pain in adults Handbook of pain assessment 2011 3 New York; London: Guilford 19 44

[B23] Fejer R Jordan A Hartvigsen J Categorising the severity of neck pain: establishment of cut-points for use in clinical and epidemiological research Pain 2005 119 176 182 10.1016/j.pain.2005.09.033 16298059

[B24] Zelman DC Hoffman DL Seifeldin R Dukes EM Development of a metric for a day of manageable pain control: derivation of pain severity cut-points for low back pain and osteoarthritis Pain 2003 106 35 42 10.1016/S0304-3959(03)00274-4 14581108

[B25] Jensen MP Smith DG Ehde DM Robinsin LR Pain site and the effects of amputation pain: further clarification of the meaning of mild, moderate, and severe pain Pain 2001 91 317 322 10.1016/S0304-3959(00)00459-0 11275389

[B26] Ware JE Jr Sherbourne CD The MOS 36-item short-form health survey (SF-36). I. Conceptual framework and item selection Med Care 1992 30 473 483 10.1097/00005650-199206000-00002 1593914

[B27] Boyd JH Weissman MM Thompson WD Myers JK Screening for depression in a community sample. Understanding the discrepancies between depression symptom and diagnostic scales Arch Gen Psychiatry 1982 39 1195 1200 10.1001/archpsyc.1982.04290100059010 7125849

[B28] Devins GM Orme CM Costello CG Binik YM Frizzell B Stam HJ Pullin WM Measuring depressive symptoms in illness populations: psychometric properties of the center for epidemiologic studies depression (CES-D) scale Psychol Health 1988 2 139 156 10.1080/08870448808400349

[B29] Radloff LS The CES-D scale: a self-report depression scale for research in the general population Appl Psychol Meas 1977 1 385 401 10.1177/014662167700100306

[B30] Ngo T Stupar M Cote P Boyle E Shearer H A study of the test-retest reliability of the self-perceived general recovery and self-perceived change in neck pain questions in patients with recent whiplash-associated disorders Eur Spine J 2010 19 957 962 10.1007/s00586-010-1289-x 20130932 PMC2899975

[B31] Hosmer DW Lemeshow S May S Applied survival analysis: regression modeling of time-to-event data 2008 2 Hoboken, New Jersey: John Wiley & Sons

[B32] Vittinghoff E Glidden DV Shiboski SC McCulloch CE Regression methods in biostatistics: linear, logistic, survival, and repeated measures models 2005 New York: Springer 133 156

[B33] Cohen J Statistical power analysis for the behavioral sciences 1988 2 Hillsdale, New Jersey: L. Erlbaum Associates 77 81

[B34] Harrell FE Jr Lee KL Mark DB Multivariable prognostic models: issues in developing models, evaluating assumptions and adequacy, and measuring and reducing errors Stat Med 1996 15 361 387 10.1002/(SICI)1097-0258(19960229)15:4<361::AID-SIM168>3.0.CO;2-4 8668867

[B35] Collett D Modelling survival data in medical research 2003 2 London: Chapman & Hall/CRC 55 169

[B36] Kerr KF McClelland RL Brown ER Lumley T Evaluating the incremental value of new biomarkers with integrated discrimination improvement Am J Epidemiol 2011 174 364 374 10.1093/aje/kwr086 21673124 PMC3202159

[B37] Altman DG Royston P What do we mean by validating a prognostic model? Stat Med 2000 19 453 473 10.1002/(SICI)1097-0258(20000229)19:4<453::AID-SIM350>3.0.CO;2-5 10694730

[B38] Hosmer DW Lemeshow S Applied logistic regression 2000 2 New York: John Wiley & Sons 160 164

[B39] Beneciuk JM Bishop MD George SZ Clinical prediction rules for physical therapy interventions: a systematic review Phys Ther 2009 89 114 124 10.2522/ptj.20080239 19095806 PMC2636674

[B40] Kamper SJ Hancock MJ Maher CG Optimal designs for prediction studies of whiplash Spine 2011 36 S268 S274 22020594 10.1097/BRS.0b013e3182388202

[B41] Carragee EJ Validity of self-reported history in patients with acute back or neck pain after motor vehicle accidents Spine J 2008 8 311 319 10.1016/j.spinee.2007.04.008 17662666

[B42] Childs JD Cleland JA Development and application of clinical prediction rules to improve decision making in physical therapist practice Phys Ther 2006 86 122 131 16386067 10.1093/ptj/86.1.122

